# Association between brachial‐ankle pulse wave velocity and cardiovascular and cerebrovascular disease in different age groups

**DOI:** 10.1002/clc.23777

**Published:** 2022-01-23

**Authors:** Da Sen Sang, Qi Zhang, Da Song, Jie Tao, Shou Ling Wu, Yong Jun Li

**Affiliations:** ^1^ Department of Internal Medicine Hebei Medical University Shijiazhuang Hebei China; ^2^ Department of Cardiology Baoding No. 1 Central Hospital Baoding Hebei China; ^3^ Department of Cardiology Kailuan General Hospital Tangshan Hebei China; ^4^ Department of Cardiology The Second Hospital of Hebei Medical University Shijiazhuang Hebei China

**Keywords:** age, brachial‐ankle pulse wave velocity, cardiovascular and cerebrovascular disease

## Abstract

**Background:**

To investigate the association between brachial‐ankle pulse wave velocity (baPWV) and cardiovascular and cerebrovascular disease (CVD) in different age groups.

**Methods:**

A total of 39 417 people, receiving Kailuan physical examination, completing baPWV examination from 2010 to 2017, with no history of CVD and atrial fibrillation, were selected as the observation objects. The population was categorized into one age group per 10 years, namely the <50, 50–59, 60–69, 70–79, and ≥80‐year‐old groups, and the total population, and each group was further assigned into three classes according to the triple quartiles of baPWV. Kaplan–Meier method helped to calculate the cumulative incidence of CVD in different age groups. The effect of baPWV on CVD in different age groups was evaluated using the Cox proportional hazards regression model.

**Results:**

Kaplan–Meier survival curve indicated statistical significance (*p* < .05) in the cumulative incidence of CVD among the whole population, <50, 50–59, and 60–69‐year‐old groups, while the cumulative incidence of end‐point events among the baPWV subgroups of 70–79 and ≥80‐year‐old groups exhibited no statistical significance (*p* > .05). Compared with baPWV in the Q1 group, hazard ratio value (95% confidence interval [CI]) of CVD in the Q3 group was 4.14 (95% CI: 2.98–5.75) in the total population, 2.98 (95% CI: 1.08–8.21) in <50‐year‐old population, 4.49 (95% CI: 2.89–7.00) in 50–59‐year‐old population, 2.78 (95% CI: 1.76–4.39) in 60–69‐year‐old population, 1.39 (95% CI: 0.86–2.24) in 70–79‐year‐old population, and 1.15 (95% CI: 0.55–2.41) in ≥80‐year‐old population.

**Conclusion:**

CVD risk attributed to increased arterial stiffness reduces with age.

## 1 INTRODUCTION

Arterial stiffness indicates the aging of blood vessels.^1^ A line of evidence confirmed that arterial stiffness is not only the result of conventional cardiovascular risk factors acting on blood vessels,^2^ but also a predisposing factor for hypertension,^3^, ^4^ diabetes,^5^, ^6^ cardiovascular events,^7^, ^8^ cognitive dysfunction,^9^, ^10^ and renal impairment.^11^ In 1989, a positive association between arterial stiffness and cardiovascular disease (CVD) events was initially reported by Tadakazu Hirai.^12^ The subsequent arterial stiffness risk in communities study,^13^ Hoorn study,^14^ Maastricht study,^15^ and chronic renal insufficiency cohort study^16^ reported a correlation between arterial stiffness and hypertension, abnormal glucose metabolism and diabetes, cognitive dysfunction, and renal impairment, respectively. Another study revealed a 6% enhancement in the risk of all‐cause mortality for every 1 cm/s increase in brachial‐ankle pulse wave velocity (baPWV).^17^

There is a linear relationship between arterial stiffness and age.^18^ Consequently, increasing age has been identified as a nonmodifiable risk factor for CVD, while the impact and magnitude of exposure to different risk factors vary among age groups. Emma F. van Bussel reported a decline in the association between some risk factors and CVD with increasing age.^19^ As an important risk factor of CVD, it is not clear whether there are differences in the effects of exposure to arterial stiffness in different ages on CVD. To address this issue, the present study analyzed the association between arterial stiffness and CVD at different ages, based on data from the Kailuan study (registration number ChiCTR‐TNC‐11001489) using baPWV as an index.

## 2 METHODS

### 2.1 Study population

From 2006 to 2007, a total of 101 510 (81 110 males and 20 400 females) in‐service and retired workers of Kailuan Group registered for the first time for health examination in Kailuan General Hospital and 11 affiliated hospitals. Thereafter, the second, third, fourth, fifth, and sixth health check‐ups were conducted in 2008–2009, 2010–2011, 2012–2013, 2014–2015, and 2016–2017, respectively. Some of the observation subjects underwent baPWV determination at the third, fourth, fifth, and sixth health check‐ups. This present study included those candidates who completed the concurrent health inspection and baPWV determination for analyses. Inclusion criteria: (i) those who underwent Kailuan physical examination in 2010–2011, 2012–2013, 2014–2015, and 2016–2017; (ii) those who completed baPWV examination at the same time; (iii) those who consented to participate in this study. Exclusion criteria: (i) those with severe physical disabilities and failed to undergo the examination; (ii) those who did not agree to participate in this study; (iii) those with a previous history of CVD, atrial fibrillation, and those with incomplete data of blood pressure. This study was conducted in accordance with the Declaration of Helsinki and was approved by the Ethics Committee of Kailuan General Hospital.

### 2.2 Baseline information

Epidemiological investigations and biochemical and anthropological measurements were detailed in the published literature.^20^ Subjects sat still for 15 min before measuring their blood pressure. A bench‐top mercurial sphygmomanometer was employed to measure the right brachial pressure. Three consecutive measurements were taken with an interval of 1–2 min between each measurement, and the average of the three measurements was considered. mean arterial pressure (MAP) = diastolic blood pressure (DBP) + 1/3PP. Body mass index (BMI) = weight (kg)/height (m)^2^. Smoking was defined as an average of ≥1 cigarette/day in the last year. Physical activity was defined as exercise ≥3 times per week, each lasting ≥30 min.

### 2.3 BaPWV determination

We collected baPWV values using a BP‐203 RPE III networked arterial stiffness detection device produced by Omron Health Medical Co., Ltd.^21^ Measurements were taken between 7:00 and 9:00 in the morning on the examination day. Participants refrained from smoking or drinking beverages for 24 h before the examination. During baPWV assessment, participants sat for more than 5 min in a room with a controlled temperature of 22–25°C. Participants wore light clothing, reclined in a supine position without a pillow and were asked to keep quiet during the examination. Both arms and ankles were wrapped in cuffs. The lower edge of the arm cuff was positioned 2–3 cm above the cubital fossa transverse striation, while the lower edge of the ankle cuff was positioned 1–2 cm above the superior aspect of the medial malleolus. Electrocardiogram electrodes were placed on both wrists, and a heartbeat monitor was placed on the left edge of the sternum. In this study, each participant underwent two measurements, and the second data was taken as the final result. The larger values of baPWV on the left and right sides were taken for analysis.

### 2.4 Follow‐up and end‐point event

After the completion of baPWV determination, that is, the starting point of follow‐up, trained medical staff reviewed the inpatient diagnosis and recorded the end‐point events of the observation objects in the Affiliated Hospitals of Kailuan Group and the Designated Hospitals for Medical and Health Insurance of China every year. The end‐point events were defined as CVD during the follow‐up, including stroke and myocardial infarction (please refer to the Standards from World Health Organization for their definitions and diagnostic criteria). Based on the inpatient medical records, professional doctors confirmed all diagnoses. The time and event of the first event were considered as the end‐point for those with ≥2 events, and the final follow‐up date for those without events was December 31, 2019.

### 2.5 Statistical analysis

The measured data followed the normal distribution and were expressed as mean ± SD and a one‐way analysis of variance was adopted for the intergroup comparison. The measurement data of nonnormal distribution were represented as M(Q1, Q3), and the study groups were compared by Kruskal–Wallis rank‐sum test. Frequency and percentage were employed to express classified variables, and the *χ* ^2^ test was performed for the intergroup comparison. The incidence of CVD and stroke in the whole population and different age groups of baPWV was computed by the Kaplan–Meier method. The log‐rank test was utilized to compare the differences of CVD and stroke between the whole population and different age groups of baPWV. Meanwhile, the Cox regression multiplication model was applied to observe the interaction between age‐based groups and baPWV tertiles. Furthermore, the present study also calculated the hazard ratio (HR) and 95% confidence interval (CI) of CVD in the whole population and different age groups (with the first percentile as the control), and the HR and 95% CI of CVD in baPWV increased by 1 SD. Owing to the high risk of mortality on cardiovascular events, the death competitive risk models were explored for patients over 70 years old. For the sensitivity analysis, a Cox regression analysis of the effect of baPWV on CVD was done after excluding study subjects with an ankle‐brachial index (ABI) < 0.9 to avoid a low (ABI) that might affect baPWV values. All data were analyzed statistically with the help of the SAS 9.4 software, and *p* < .05 was considered a statistically significant difference (two‐sided).

## 3 RESULTS

### 3.1 Characteristics of the study population

A total of 41 121 people who received physical examination for baPWV determination in Kailuan General Hospital from 2010 to 2017 were considered as research objects. The present study collectively excluded 523 cases with a previous history of CVD, 226 with a history of atrial fibrillation, and 955 with incomplete blood pressure data, and finally included 39 417 cases for statistical analyses. The study population encompassed 28 571 (72.48%) males and 10 846 (27.52%) females, with a mean age of 51.65 ± 13.28 years and a mean baPWV of 1602.43 ± 362.70 cm/s. The enrolled subjects were classified into three groups by trichotomies based on baPWV, and statistically significant differences were observed in age, gender, baPWV, SBP, DBP, MAP, triglyceride, high sensitivity C reactive protein, total cholesterol, high‐density lipoprotein cholesterol, low‐density lipoprotein cholesterol, fasting blood glucose, BMI, hypertension, smoking habit, physical exercise, diabetes, and antihypertensive medication among the three groups (*p* < .05; Table 1).

**Table 1:** Baseline characteristics of the study population

Parameters	Overall	Q1	Q2	Q3	*p* Value
Male (*n* [%])	28 571 (72.48)	7272 (55.40)	10 529 (80.10)	10 770 (81.93)	<.001
Age (years)	51.65 ± 13.28	44.09 ± 10.65	51.65 ± 13.28	60.33 ± 12.68	<.001
baPWV (cm/s)	1602.43 ± 362.70	1167.39 ± 105.96	1418.50 ± 69.91	1850.90 ± 301.04	<.001
SBP (mmHg)	130.52 ± 20.92	118.71 ± 18.84	130.09 ± 16.41	143.07 ± 19.81	<.001
DBP (mmHg)	81.89 ± 10.94	76.21 ± 9.23	82.73 ± 9.78	86.75 ± 11.04	<.001
MAP (mmHg)	98.14 ± 12.87	90.38 ± 10.85	98.51 ± 10.76	105.52 ± 12.19	<.001
HR (bpm)	74.68 ± 11.15	72.76 ± 10.07	74.31 ± 10.68	76.70 ± 12.19	<.001
TG^a^	1.8	1.43	1.9	2.1	<.001
	(0.50–2.20)	(0.73–1.60)	(0.91–2.10)	(1.01–2.37)	
hsCRP^a^	2.06	1.69	1.96	2.52	<.001
	(0.50–2.20)	(0.40–1.90)	(0.50–2.20)	(0.60–2.73)	
TC (mmol/L)	4.97 ± 1.52	4.74 ± 1.42	5.03 ± 1.42	5.14 ± 1.28	<.001
HDL‐C (mmol/L)	1.45 ± 0.73	1.46 ± 0.63	1.43 ± 0.75	1.46 ± 0.72	.001
LDL‐C (mmol/L)	2.74 ± 0.78	2.56 ± 0.98	2.76 ± 0.89	2.83 ± 1.01	<.001
FBG (mmol/L)	5.80 ± 2.11	5.22 ± 1.29	5.70 ± 2.10	6.49 ± 2.52	<.001
BMI (kg/mm^2^)	25.00 ± 3.45	24.28 ± 3.49	25.27 ± 3.38	25.42 ± 3.38	<.001
Hypertension (*n* [%])	15 410 (39.09)	1679 (10.90)	4756 (30.86)	8975 (58.24)	<.001
Smoking habit (*n* [%])	14 087 (35.74)	3725 (28.38)	5391 (41.01)	4971 (37.81)	<.001
Physical exercise (*n* [%])	4433 (11.25)	1219 (9.29)	1551 (11.80)	1663 (12.65)	<.001
Diabetes (*n* [%])	5156 (13.08)	445 (3.39)	1280 (9.74)	3431 (26.10)	<.001
Antihypertensive medication (*n* [%])	3747 (9.51)	244 (1.86)	925 (7.04)	2578 (19.61)	<.001

Abbreviations: baPWV, brachial‐ankle pulse wave velocity; BMI, body mass index; DBP diastolic blood pressure; Fbg, fasting blood glucose; HDL‐C, high‐density lipoprotein cholesterol; HR, heart rate; hsCRP, high sensitivity C reactive protein; LDL‐C, low‐density lipoprotein cholesterol; MAP, mean arterial pressure; SBP, systolic blood pressure; TC, total cholesterol; TG, triglyceride.

^a^ Expressed in M(Q1–Q3).

### 3.2 Cumulative incidence rates of CVD

**Figure 1:**
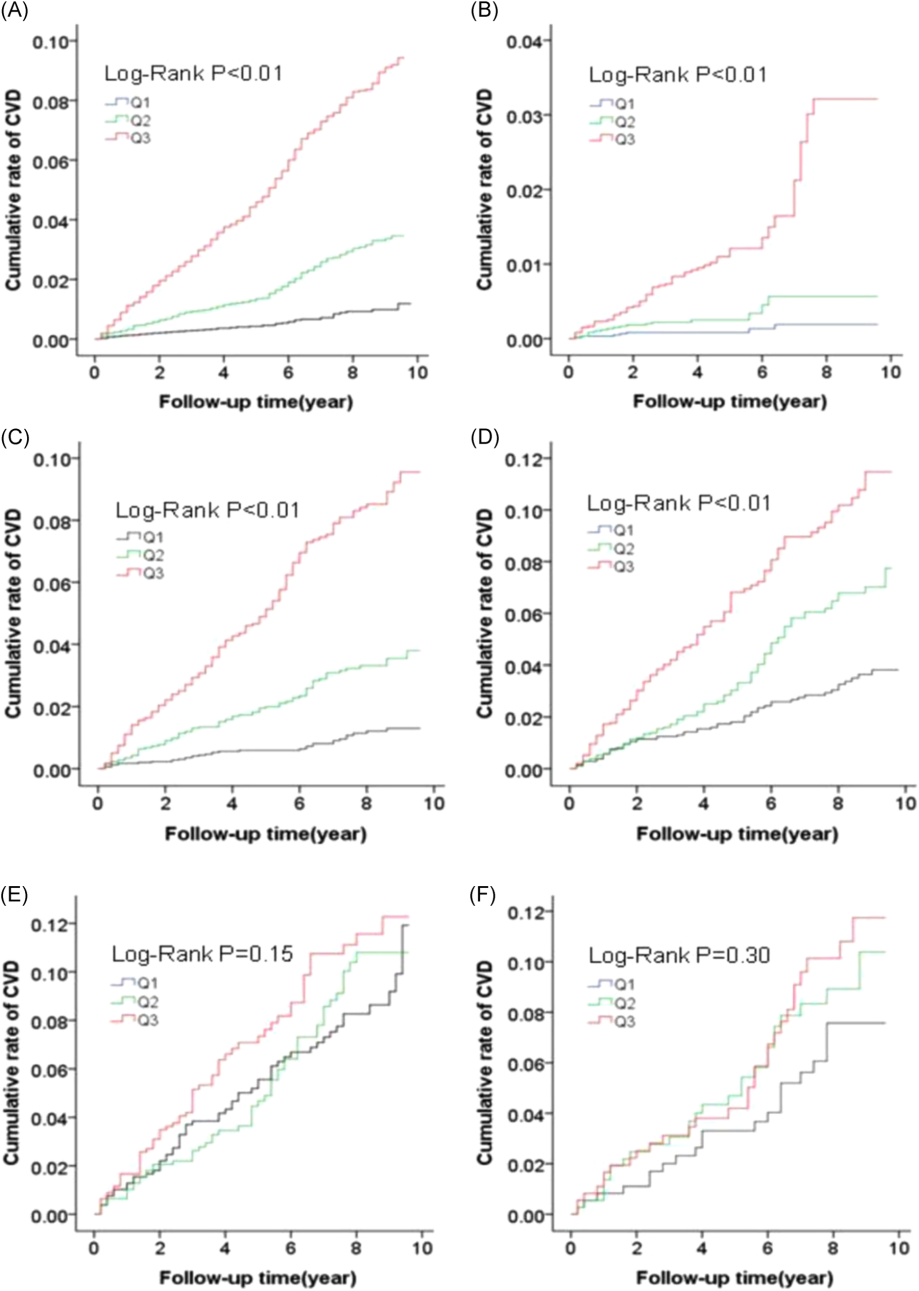
(A) The whole population; (B) <50‐year‐old; (C) 50–59‐year‐old; (D) 60–69‐year‐old; (E) 70–79‐year‐old; (F) ≥ 80‐year‐old. CVD, cardiovascular and cerebrovascular disease

With a mean follow‐up of 5.12 ± 2.37 years, 986 cases of CVD were reported, with cumulative incidence rates of 0.12%, 3.46%, and 9.43% in the Q1, Q2, and Q3 quartile groups, respectively, in the total population. A statistically significant difference was confirmed by the log‐rank test in the cumulative incidence between the groups (*p* < .05). Kaplan–Meier survival analysis revealed that the cumulative incidence of CVD was 0.19%, 0.56%, and 3.22% in the subgroup <50‐year‐old from Q1 to Q3 group, 1.30%, 3.80%, and 9.56% in 50–59‐year‐old group, 3.82%, 7.74%, and 11.47% in 60–69‐year‐old group, 11.93%, 10.80%, and 12.28% in 70–79‐year‐old group, and 7.58%, 10.39%, and 11.74% in the subgroup ≥80‐year‐old, respectively. Based on the log‐rank test, the cumulative incidence of end‐point events exhibited statistically significant difference among the subgroups of <50, 50–59, and 60–69‐year old groups (*p* < .05), while the difference was statistically insignificant in the cumulative incidence of end‐point events among the subgroups of 70–79 and ≥80‐year old groups (*p* = .15 and *p* = .30, respectively; see Figure 1).

### 3.3 Cox proportional‐hazards model affecting CVD

Considering CVD as the dependent variable, baPWV tertile as independent variables, and Q1 tertile group as the control group, age, gender, heart rate, BMI, TC, MAP, fasting glucose, high‐sensitivity C‐reactive protein, smoking, physical activity, and whether taking antihypertensive drugs were corrected. Cox regression analysis confirmed that the age and baPWV group had interaction with CVD (*p* for interaction < .001). Compared to baPWV in the Q1 group, the HR values (95% CI) of CVD in the Q3 group were 4.14 (95% CI: 2.98–5.75), 2.98 (95% CI: 1.08–8.21), 4.49 (95% CI: 2.89–7.00), 2.78 (95% CI: 1.76–4.39), 1.39 (95% CI: 0.86–2.24), and 1.15 (95% CI: 0.55–2.41) in the total population, <50‐year‐old population, 50–59‐year‐old population, 60–69‐year‐old population, 70–79‐year‐old population, and ≥80‐year‐old population, respectively. Relative to the Q1 group, the HR values (95% CI) of stroke in the Q3 group were 4.22 (95% CI: 3.04–5.86) in the total population, 3.80 (95% CI: 3.04–5.86) in <50‐year‐old group, 4.54 (95% CI: 2.91–7.06) in the 50–59‐year‐old group, 3.13 (95% CI: 2.11–4.66) in the 60–69‐year‐old group, 1.61 (95% CI: 0.96–2.69) in the 70–79‐year‐old group, and 0.86 (95% CI: 0.38–1.92) in ≥80‐year‐old group. By every one increase of SD of baPWV, the HR values (95% CI) of CVD were 1.30 (95% CI: 1.25–1.36), 1.44 (95% CI: 1.22–1.70), 1.21 (95% CI: 1.08–1.36), 1.32 (95% CI: 1.14–1.53), 1.08 (0.97–1.20), and 1.13 (95% CI: 0.77–1.66) in the total population, <50, 50–59, 60–69, 70–79, and ≥80‐year‐old groups, respectively, whereas the HR values (95% CI) of stroke events were 1.28 (95% CI:1.23–1.34), 1.40 (95% CI: 1.21–1.68), 1.26 (95% CI: 1.21–1.31), 1.24 (95% CI: 1.10–1.41), 1.27 (95% CI: 1.08–1.47), and 0.92 (95% CI: 0.65–1.32), respectively (Table 2). The death competitive risk model of partakers over 70 years old was analyzed, and the results were found to be consistent (see attached Table 1).

**Table 2:** Cox proportional‐hazards model affecting CVD

Groups	CVD events			Stroke events		
	No. of event/no. of subjects	HR (95% CI)^a^	*p* Value	No. of event/no. of subjects	HR (95% CI)^a^	*p* Value
Overall	986/39 417			818/39 417		
Q1	73/13 127			55/13 127		
Q2	232/13 144	2.02 (1.44–2.83)	<.01	188/13 144	2.03 (1.45–2.87)	<.01
Q3	681/13 146	4.14 (2.98–5.75)	<.01	575/13 146	4.22 (3.04–5.86)	<.01
Per + 1 SD		1.30 (1.25–1.36)	<.01		1.28 (1.23–1.34)	<.01
<50 years	96/17 941			92/17 941		
Q1	7/5988			7/5988		
Q2	18/5970	1.17 (0.38–3.56)	.78	16/5970	1.17 (0.39–3.57)	.78
Q3	71/5983	2.98 (1.08–8.21)	.03	69/5983	3.80 (1.40–10.33)	.01
Per + 1 SD		1.44 (1.22–1.70)	<.01		1.40 (1.21–1.68)	<.01
50–59 years	352/11 890			338/11 890		
Q1	35/3957			33/3957		
Q2	99/3965	2.25 (1.41–3.59)	<.01	96/3965	2.27 (1.42–3.61)	<.01
Q3	218/3968	4.49 (2.89–7.00)	<.01	209/3968	4.54 (2.91–7.06)	<.01
Per + 1 SD		1.21 (1.08–1.36)	<.01		1.26 (1.21–1.31)	<.01
60–69 years	293/6244			242/6244		
Q1	58/2081			43/2081		
Q2	93/2080	2.27 (1.51–3.43)	<.01	79/2080	2.05 (1.38–3.04)	<.01
Q3	142/2083	2.78 (1.76–4.39)	<.01	120/2083	3.13 (2.11–4.66)	<.01
Per + 1 SD		1.32 (1.14–1.53)	<.01		1.24 (1.10–1.41)	<.01
70–79 years	168/2278			141/2278		
Q1	52/761			39/761		
Q2	53/758	1.04 (0.65–1.66)	.42	47/758	1.27 (0.76–2.11)	.36
Q3	63/759	1.39 (0.86–2.24)	.26	55/759	1.61 (0.96–2.69)	.67
Per + 1 SD		1.08 (0.97–1.20)	.14		1.27 (1.08–1.47)	.01
≥80 years	77/1064			66/1064		
Q1	21/354			21/354		
Q2	26/355	1.27 (0.63–2.54)	.28	23/355	1.23 (0.59–2.53)	.58
Q3	30/355	1.15 (0.55–2.41)	.08	22/355	0.86 (0.38–1.92)	.71
Per + 1 SD		1.13 (0.77–1.66)	.81		0.92 (0.65–1.32)	.67

Abbreviations: CI, confidence interval; CVD, cardiovascular disease; HR, hazard ratio.

^a^ Adjusted for baseline age, sex, heart rate, body mass index, fasting blood glucose, total cholesterol, high sensitivity C reactive protein, mean arterial pressure, smoking habit, physical exercise, antihypertensive medication.

### 3.4 Sensitivity analysis

The sensitivity analysis was conducted, excluding those with an ABI < 0.9. The results of this analysis were in agreement with the previous results, substantiating the elevated risk of cardiovascular and stroke events with increasing baPWV in the total population, in the <50, 50–59, and 60–69‐year‐old groups. Nonetheless, the association between baPWV and CVD and stroke events was not statistically significant (*p* > .05) in the 70–79 and ≥80‐year‐old groups (Table 3).

**Table 3:** Cox proportional‐hazards model (sensitivity analysis) affecting CVD

Groups	CVD events			Stroke events		
	No. of event/no. of subjects	HR (95% CI)^a^	*p* Value	No. of event/no. of subjects	HR (95% CI)^a^	*p* Value
Overall	590/37 917			499/37 917		
Q1	40/12 647			35/12 647		
Q2	136/12 629	2.28 (1.58–3.29)	<.01	86/12 629	2.13 (1.46–2.89)	<.01
Q3	414/12 641	4.36 (3.06–6.21)	<.01	378/12 641	4.33 (3.13–6.09)	<.01
Per + 1 SD		1.36 (1.28–1.46)	<.01		1.30 (1.24–1.47)	<.01
<50 years	53/17 210			48/17 210		
Q1	3/5739			2/5999		
Q2	11/5743	1.97 (0.53–7.37)	.31	9/5743	1.15 (0.33–3.42)	.34
Q3	39/5728	4.12 (1.18–14.41)	.03	37/5728	3.35 (1.42–7.89)	.01
Per + 1 SD		1.56 (1.38–1.76)	<.01		1.41 (1.20–1.69)	<.01
50–59 years	223/11 601			201/11 601		
Q1	24/3867			20/3867		
Q2	63/3870	2.29 (1.42–3.70)	<.01	55/3870	2.22 (1.40–3.41)	<.01
Q3	136/3864	4.64 (2.96–7.27)	<.01	126/3864	3.98 (1.97–5.12)	<.01
Per + 1 SD		1.28 (1.19–1.37)	<.01		1.24 (1.20–1.32)	<.01
60–69 years	160/5971			125/5971		
Q1	28/1991			23/1991		
Q2	58/1991	2.13 (1.33–3.41)	<.01	40/1991	2.13 (129–3.14)	<.01
Q3	74/1989	2.76 (1.70–4.48)	<.01	62/1989	3.03 (2.01–4.45)	<.01
Per + 1 SD		1.32 (1.14–1.53)	<.01		1.22 (1.11–1.35)	<.01
70–79 years	99/2158			80/2158		
Q1	28/718			20/718		
Q2	33/721	1.09 (0.65–1.83)	.75	27/721	1.31 (0.68–2.45)	.28
Q3	38/719	1.29 (0.75–2.22)	.35	33/719	1.47 (0.79–2.48)	.56
Per + 1 SD		1.14 (0.92–1.39)	.24		1.28 (1.09–1.40)	.01
≥80 years	55/977			45/977		
Q1	13/325			10/325		
Q2	23/326	1.53 (0.75–3.10)	.24	20/326	1.46 (0.78–3.04)	.35
Q3	19/326	1.34 (0.64–2.84)	.44	15/326	1.56 (0.72–3.45)	.50
Per + 1 SD		1.07 (0.80–1.44)	.63		0.98 (0.66–1.35)	.60

Abbreviations: CI, confidence interval; CVD, cardiovascular disease; HR, hazard ratio.

^a^ Adjusted for baseline age, sex, heart rate, body mass index, fasting blood glucose, total cholesterol, high sensitivity C reactive protein, mean arterial pressure, smoking habit, physical exercise, antihypertensive medication.

## 4 DISCUSSION

The present study established arterial stiffness as a risk factor for cardiovascular events, and the risk of both total CVD and stroke aggravated with increased arterial stiffness. It was also claimed that such an increased risk might be age‐related; as is, the risk of CVD due to increased arterial stiffness tended to decrease with increasing age.

Previous studies revealed that increased arterial stiffness is a predisposing factor for arteriosclerotic cardiovascular disease, heart failure, kidney damage, and cognitive impairment, whether measured by carotid‐femoral pulse wave velocity (cfPWV) or baPWV.^2^, ^22^, ^23^, ^24^, ^25^ Ohkuma et al.^26^ found that for every 1 SD of baPWV in 14 673 people without CVD, the risk of CVD increased by 1.19 times. Notably, we realized a dose‐dependent increase in the risk of CVD attributed to enhanced arterial stiffness, with a 30% and 28% increase in CVD and stroke risk, respectively, for every 1 SD increase in baPWV (339.43 cm/s) in the total population. However, in different age groups, there was a reduction in the risk of CVD and stroke per 1 SD increase with increasing age. The risk values for CVD and stroke declined from 1.44 (95% CI: 1.22–1.70) and 1.40 (95% CI: 1.21–1.68) in the <50‐year‐old group to 1.08 (95% CI: 0.97–1.20) and 0.92 (95% CI: 0.6–1.32) in the 70‐ and 80‐year‐old groups, respectively. Therefore, the risk due to increased baPWV was suggested to be age‐dependent. Furthermore, the risk of CVD and stroke in Q3 in the <50‐year‐old group was lower than that in Q3 in the 50–59‐year‐old group; however, no statistical difference in the increase of risk in Q3 in the over 70‐year‐old group was evident. The trend toward a lower risk of CVD and stroke in the Q3 group with increasing age was less pronounced and may be related to the lower number of CVD and stroke events in the group  <50‐year‐old.

The presence of lower limb arterial occlusive lesions contributed to the inaccuracy in the measured baPWV values.^27^ Lower limb arterial occlusive lesion was also a risk factor for CVD and all‐cause mortality.^28^, ^29^ Henceforth, we repeated the main analysis except for those with an ABI < 0.9, and the results remained unchanged. Moreover, given the high mortality rate in older individuals and the potential for competing risks of death, the main results of the analysis using the competitive risks of death model in the group over 70 years old also remained unaltered, which ensured the reliability of the present results. No literature was available on the correlation between baPWV and CVD in people (those with ABI < 0.9 were excluded) using death‐competitive risk models.

The present study concluded that increased arterial stiffness‐induced risk of CVD and stroke decreased or even disappeared with age, but the mechanism perplexed us. Younger individuals exhibit fewer risk factors for CVD compared to older individuals, and the increased arterial stiffness, as a single risk factor, contributes more to CVD and stroke than older individuals, whereas older individuals have more cardiovascular risk factors and the synergistic effect of multiple risk factors may diminish the risk effect of single arterial stiffness. Another possible mechanism is the so‐called “ceiling” effect.

Our findings emphasized higher risk due to arterial stiffness in younger individuals, which is independent of traditional risk factors. These results significantly claimed the potential of baPWV determination as a screening method for younger individuals at risk. The geriatric population should also undergo routine baPWV testing. Prolonged exposure to multiple cardiovascular risk factors results in increased arterial stiffness, justifying the necessity for the regulation of multiple risk factors in older individuals with increased arterial stiffness. Moreover, in the absence of effective drugs to improve arterial stiffness, it is thus imperative to maintain a healthy lifestyle and control risk factors, including hypertension, at the individual or group level to reduce or delay the progression of arterial stiffness.

## 5 LIMITATIONS

Despite the large sample size observed in this study and the adjustment for various confounding factors to ensure the reliability of the results, there are several limitations to our study. First, a larger proportion of males observed in this study thus indicated the presence of selection bias. Second, arterial stiffness was measured using baPWV rather than the gold standard cfPWV.^30^ However, baPWV could serve as an alternative to cfPWV owing to its simple operation, high repeatability, and similar value in predicting clinical prognosis, which encourages its wide application in Asian populations.^31^ Furthermore, insulin resistance, common in hypertensive patients, wields an independent role for baPWV but not for cfPWV,^32^, ^33^, ^34^ and therefore, the American Heart Association emphasizes that these indicators are not necessarily interchangeable.^30^

## 6 CONCLUSION

The arterial stiffness is an independent risk factor for compound cardiovascular events, however, the risk of CVD due to increased arterial stiffness tended to decrease with increasing age.

## CONFLICT OF INTERESTS

The authors declare that there are no conflict of interests.

## Supporting information

Supplementary information.

## Data Availability

No data are available. The datasets used and/or analyzed during the current study are available from the corresponding author on reasonable request.
