# Supplementary material for: Association between brachial‐ankle pulse wave velocity and cardiovascular and cerebrovascular disease in different age groups
Source: Clin Cardiol. 2022 Jan 23;45(3):315–23. doi: 10.1002/clc.23777 (PMC8922528; doi:10.1002/clc.23777)
Supplement: Supplementary file 1 — Supplementary information. [file CLC-45-315-s001.docx]

**Attached Table 1 Cox proportional-hazards model (death competitive risk model) affecting CVD**

| **Groups** | **CVD events** | | | **Strock events** | | |
| --- | --- | --- | --- | --- | --- | --- |
|  | No of Event/No of Subjects | HR(95%CI)* | P value | No of Event/No of Subjects | HR(95%CI)* | P value |
| **70-79years** | 174/2350 |  |  | 141/2350 |  |  |
| **Q1** | 52/781 |  |  | 39/781 |  |  |
| **Q2** | 56/786 | 0.94(0.64-1.37) | 0.74 | 47/786 | 1.07(0.70-1.65) | 0.36 |
| **Q3** | 66/783 | 1.05(0.71-1.55) | 0.82 | 55/783 | 1.19(0.76-1.85) | 0.67 |
| **Per+1SD** |  | 1.04(0.93-1.17) | 0.21 |  | 1.25(1.05-1.37) | 0.01 |
| **≥80years** | 77/1095 |  |  | 66/1095 |  |  |
| **Q1** | 22/365 |  |  | 21/365 |  |  |
| **Q2** | 26/365 | 1.25(0.68-2.30) | 0.47 | 23/365 | 1.25(0.68-2.30) | 0.47 |
| **Q3** | 29/365 | 1.11(0.59-2.11) | 0.75 | 22/365 | 1.11(0.59-2.11) | 0.75 |
| **Per+1SD** |  | 1.15(0.78-1.77) | 0.78 |  | 0.98(0.69-1.44) | 0.58 |

*is adjusted for baseline age, sex, heart rate, BMI, Fbg, TC, hsCRP, MAP, Smoking habit, Physical exercise, Antihypertensive medication.
